# Antibacterial activity of the biogenic volatile organic compounds from three species of bamboo

**DOI:** 10.3389/fpls.2024.1474401

**Published:** 2024-11-15

**Authors:** Yifan Duan, Bingyang Lv, Chunlong Zhang, Lisha Shi, Jingting Li, Yanjun Liu, Qibing Chen

**Affiliations:** ^1^ College of Art, Sichuan Tourism University, Chengdu, China; ^2^ College of Landscape Architecture, Sichuan Agricultural University, Chengdu, China; ^3^ College of Fine Art and Calligraphy, Sichuan Normal University, Chengdu, China; ^4^ School of Business, Sichuan University Jinjiang College, Meishan, China

**Keywords:** anti-microbial, BVOCs, phyllostachys eduli, recreational forests, SPME-GC/MS

## Abstract

Plant biogenic volatile organic compounds (BVOCs) possess ecological functions in antimicrobial benefits and air purification. The objectives of the study were to determine the differences in antimicrobial capacity of bamboo forests at different sampling sites. Three common bamboo species—*Phyllostachys edulis, Bambusa emeiensis*, and *Phyllostachys violascens*—were selected to determinate the antimicrobial activity of bamboo forests as well as under *ex vivo* conditions. Natural sedimentation method was used to determine the microbe counts in bamboo forests, and the microbe counts in grassland in the same area was measured as control treatment. The results showed that except for the *P. violascens* in Ya’an, the airborne microbial content of the sampling sites in bamboo forests was significantly lower relative to that of grassland in the same area, and inhibition rate reached 74.14% in the *P. violascens* forest in Dujiangyan. *P. edulis* forest and *P. violascens* forest in Ya'an had significantly lower inhibition rates than the other sampling sites, and there was no significant difference in the inhibition rates among the rest of the bamboo forest. The bacterial inhibition rate of bamboo leaves under *ex vivo* conditions varied with bamboo species and bacterial strains, with higher antibacterial activity against Gram-negative bacteria overall. *Escherichia coli* was sensitive to *B. emeiensis* leaves, while *Staphylococcus aureus* and *Bacillus subtilis* were sensitive to *P. violascens* leaves. Moreover, *Candida albicans, S. cremoris*, and *Shigella Castellani* were sensitive to *P. edulis* leaves. An analysis of the BVOCs composition from *P. edulis* collected in Changning by SPME-GC/MS revealed that the relative content of ocimene was obviously higher than other components. This study showed that *P. edulis* BVOCs have strong inhibitory ability to the tested microorganisms, and its main constituent, ocimene, has health benefit. *P. edulis* has the potential to become a forest recreation bamboo species.

## Graphical abstract

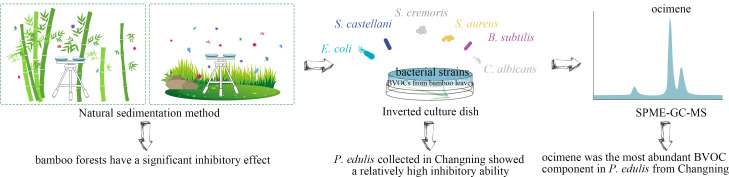

## Highlights

- Bamboo forests have strong antimicrobial capacity against microorganisms relative to grasslands.
- Bamboo growth status influences the BVOC inhibition capacity significantly.
- Stronger antibacterial activity of BVOCs from bamboo leaves against Gram-negative than Gram-positive bacteria.
- Overall, *Phyllostachys edulis* had a stronger and wider range antibacterial effect among the three bamboo species.
- Terpenes are the main components in *Phyllostachys edulis* BVOCs.

## 1 Introduction

Plant biogenic volatile organic compounds (BVOCs) are low-boiling point and complex mixtures of organic compounds synthesized and released by plants (Schimitberger et al., 2018). These compounds are predominately terpenes, phenols, aldehydes, alcohols, among others (Penuelas and Llusia, 2003). The release of plant BVOCs is influenced by both plant intrinsic and environmental factors. Temperature and light are the main environmental factors affecting BVOC release, with the highest release rates generally observed during summer (Wolfertz et al., 2003; Motonori et al., 2018). BVOCs contribute to ecological benefits because of its significant inhibitory and bactericidal effects on specific plant pathogens and human pathogens (Sharifi et al., 2018; Božik et al., 2017). Terpenoids are the main antibacterial active components in BVOCs, and monoterpene 3-carene could inhibit the growth of *Pseudomonas aeruginosa*, damage the normal cell morphology, leading to bacterial cell death by the destruction of cell membrane (Liu et al., 2019). Sesquiterpenes caryophyllene showed strong bacteriostatic effects against *Escherichia coli*, *Bacillus subtilis*, *Micrococcus tetragenus*, *B. thermosphacta* (Shu et al., 2020). Besides, aldehydes also have significant antibacterial effects, and studies have shown that cinnamaldehyde has antibacterial effects on pathogenic bacteria such as *Escherichia coli* and *Staphylococcus aureus* (Sanla-Ead et al., 2012; Labib et al., 2017). Benzaldehyde have been reported to cause morphological and ultrastructural changes in the cells of *Rhodobacter sphaeroides*, resulting in a bacteriostatic effect (Tahir et al., 2017). Additionally, BOVCs has direct benefits for human health. Inhaling BVOCs like limonene and pinene can result in antioxidant and anti-inflammatory effects on the airways, and the pharmacological activity of some terpenes absorbed through inhalation may be also beneficial to promote brain functions by decreasing mental fatigue, inducing relaxation, and improving cognitive performance and mood (Joung et al., 2015; Antonelli et al., 2020). However, the presence of some harmful components such as paraxylene, dichloromethane, and trichloromethane has also been detected in some plant BVOCs (Ronald, 1997; Li et al., 2022).

Bamboo is an abundant forest resources with wide distribution, large biomass, rapid growth and metabolism, and long growing periods (Lima et al., 2012; Buziquia et al., 2019; Xu et al., 2020). More than 1500 species of bamboo inhabit 22 million hectares worldwide with China accounting for 20% of the total area of bamboo globally (Zhou et al., 2017). Bamboo leaves are rich in a variety of bioactive components, including flavonoids, polysaccharides, amino acids, phenolic acids, volatile oils, etc (Shen et al., 2022; Wang et al., 2015), and have attracted much attention for their pharmacological activities such as antioxidant and antimicrobial (Liao et al., 2012; Wang et al., 2021; Zhang et al., 2005). Several classes of antimicrobial compounds have been described as responsible for antibacterial potential in bamboos, including benzoquinone (Nishina and Uchibori, 1991), chitin-binding peptides (Fujimura et al, 2005), fatty acids, such as linoleic acid (Soumya et al., 2014), and phytosterols (Tanaka et al., 2013). Studies have shown that essential oils extracted from bamboo possess a variety of active constituents with significant antibacterial effects on environmentally common fungi like yeast and bacteria including Gram-positive (*Staphylococcus aureus*, *Staphylococcus epidermidis* and *Bacillus subtilis*) and Gram-negative (*Shigella flexneri*, *Proteus vulgaris* and *Escherichia coli*) bacterial strains (Das et al., 2020; Jin et al., 2011; Shen et al., 2014). Therefore, bamboo forests not only have high economic and ecological values, but are also potentially suitable sites for air purification and healing (Zhou et al., 2005). The ability of plant BVOCs to inhibit bacterial growth depends on their composition as well as their release rate (Aguiar et al., 2014; Ben Hsouna et al., 2014; Krumal et al., 2015). It has been reported that essential oils containing mainly aldehydes or phenols, such as cinnamaldehyde, citral, carvacrol, eugenol, or thymol were characterized by the highest antibacterial activity, followed by essential oils containing terpene alcohols (Dorman and Deans, 2000; Davidson and Taylor, 2013).The composition of BVOCs released by different bamboo species differ (Motonori et al., 2018; Crespo et al., 2013), so there may be differences in the inhibitory capacity. Current studies on the bacteriostatic activity of BVOCs in bamboo focus mostly on the bacteriostatic effect of extracted essential oils. Although essential oils do contain BVOCs, not all BVOCs spontaneously released by plants are present in essential oils, and some molecules included in essential oils are artefacts of distillation (Senatore, 2000; Najar et al., 2020). Therefore, the bacteriostatic effect of essential oils do not fully reflect the bacteriostatic effect of spontaneously released BVOCs from living plants.*Phyllostachys edulis* and *Bambusa emeiensis* are the main native bamboo species in China and widely distributed. Their leaves have been reported to contain antibacterial active substances (Motonori et al., 2018; Yuan et al., 2020; Shen et al., 2024). *Phyllostachys violascens* is commonly introduced and cultivated in China but the antimicrobial activity has not been reported. In order to compare the differences in the inhibitory effects of BVOCs on environmental microorganisms between different bamboo species and different bamboo forest types during natural growth, the present study selected *Phyllostachys edulis* and *Bambusa emeiensis* and *Phyllostachys violascens* as research subjects. By comparing the inhibitory effect of BVOCs spontaneously released on environmental microorganisms in bamboo forests and bamboo leaves in *ex vivo* conditions, we investigated the differences in the inhibitory activity of different bamboo species and determination of BVOCs composition of the bamboo species with the best inhibitory effect.This study provided a scientific basis for planting configurations in recreational bamboo forests.

## 2 Materials and methods

### 2.1 General overview of the study area

Sichuan Province in China has a warm climate, abundant rainfall, and a long growing season, making most of the area suitable for bamboo growth. The experimental sample plots were selected from natural bamboo landscape forests and planted bamboo ecological gardens located in Sichuan, between longitude 103°03’–103°50’E and latitude 28°28’–30°07’N, with the bamboo forest area exceeding 10 ha. There were a total of seven sample plots, and the location information is shown in **Table 1**, involving three bamboo species, *P. edulis*, *B. emeiensis*, and *P. violascens* (**Table 1**).

**Table 1:** Distribution and geographic location of the sample plots of the three bamboo species for the bacterial suppression experiment.

Bamboo Species	Location	East Longitude; North Latitude	Area (ha)
*Phyllostachys edulis*	Yucheng District, Ya’an City	103°4′24″E; 30°10′5″N	20
	Changning County, Yibin City	105°0′23″E; 28°28′23″N	12000
*Bambusa emeiensis*	Yucheng District, Ya’an City	103°2′5″E; 29°54′19″N	15
	Muchuan County, Leshan City	103°57′57″E; 28°52′35″N	5000
*Phyllostachys violascens*	Puyang Town, Dujiangyan Irrigation Project City	103°38′60″E; 31°2’22″N	30
	Yucheng District, Ya’an City	103°3′17″E; 30°7′60″N	15
	Pujiang County, Chengdu City	103°19′19″E; 30°15′24″N	20

### 2.2 Determination of the antibacterial inhibition rates in bamboo forest environments

The natural sedimentation method was used to determine the microbial counts in bamboo forest environments and control grassland. The antibacterial inhibition rate in bamboo forest environments was calculated based on the microbial count in the bamboo forest and control grassland environments (Formulas 1 and 2). The sampling time was from 9:00 AM to 11:00 AM from July to August to minimize the effect of differences in light and temperature on the experimental results. Sampling periods with clear and windless weather were selected, and sampling points were set up in areas with minimal human interference in bamboo forests and adjacent grasslands. A total of five sampling points for each plot were randomly selected, with three replicates per sampling point. Petri dishes containing beef extract peptone medium (BPY medium) were placed horizontally on a support plate 1.5 m above the ground, and the medium was exposed to air for 15 min by opening the lid of the petri dish, then the lid of the dish was closed and sealed with a sealing film. After labeling, the plates were returned to the laboratory and incubated at a constant temperature of 37°C for 24 h, and the petri dish colonies were counted. The number of airborne microorganisms was calculated using the Omelensky formula:


(1) E=1000 N /(A/100×t×10/5) =50000 N/At

where E is the number of bacteria per unit volume of air (CFU/m^3^), A is the area in the petri dish (cm^2^), t is the sampling time (min), and N is the average number of colonies at each sampling point after incubation (pcs).

The formula for calculating the antibacterial inhibition rate:


(2) Antibacterial inhibition rate=(N2−N1)/N2×100%

where N2 is the number of microorganisms in the air of the control grassland, and N1 is the number of microorganisms in the bamboo forest environment.

### 2.3 Determination of the antibacterial effect of volatile organic compounds from bamboo leaves

#### 2.3.1 Activation culture of test strains

The bacteriostatic activity of BVOCs from bamboo leaves was determined using the method described by Krumal et al. (2015). Common pathogenic microorganism in environment, which can cause various diseases were selected including Gram-negative *Escherichia coli*, *Shigella Castellani*, Gram-positive *Staphylococcus cremoris*, *Staphylococcus aureus*, *Bacillus subtilis*, and fungi *Candida albicans*. All strains were purchased from Boulder Vanguard Ltd. Bacterial strains were inoculated with BPY medium using the scribing method and activated for 24 h at 37°C in an incubator. *Candida albicans* was inoculated on potato glucose agar (PDA) medium and activated for 48 h in an incubator at 28°C.

#### 2.3.2 Preparation of test strain suspensions

The activated bacterial strains were gently scraped off with an inoculating ring, which was immersed into a triangular flask of BPY liquid medium. After the strain was placed freely into the liquid medium, the mixture was sealed and shaken gently in a 37°C shaker constant temperature shock culture. The absorbance was measured at 600 nm, and OD_600_ = 0.3 was used as the standard bacterial solution. The activated *C. albicans* were inoculated into a triangular flask of potato dextrose broth using the same method. After sealing, they were placed in a table concentrator at 28°C for constant temperature shaking and cultivation. Their absorbance was measured at a wavelength of 560 nm, and OD_560_ = 0.3 was used as the standard bacterial liquid culture. The standard bacterial solution was prepared as a bacterial suspension containing about 10^5^ CFU·mL^−1^ using the dilution method, which serves as the test bacterial suspension.

#### 2.3.3 Collection and treatment of plant leaves

After determining the environmental bacterial inhibition rate of the bamboo forests, bamboo leaves were collected and returned to the laboratory for *ex vivo* bacterial inhibition testing. Five sample plots were randomly selected in each bamboo forest distant from anthropogenic disturbances, and branches and leaves with good and uniform growth conditions were collected with high pruning shears, stored in a constant temperature box at 4°C, and transported to the laboratory on the same day. After returning the samples to the laboratory, mature and fresh leaves of an appropriate size (without insect spots) were cut, removed from the petiole, and rinsed three times with sterile distilled water to remove surface impurities from the bamboo leaves. After rinsing the leaves, an absorbent paper was used to absorb surface moisture from the leaves. Then, degreased cotton dipped in 75% alcohol was used to remove surface residues from the leaves. Subsequently, the leaves were rinsed with sterile distilled water three times, the surface moisture was removed using absorbent paper, and the leaves were used to measure the antibacterial inhibition rate.

#### 2.3.4 Determination of the antibacterial activity in the presence of volatile substances from bamboo leaves

A volume of 0.2 mL of the prepared suspension was used to test each bacteria (*E. coli*, *Shigella Castellani*, *S. cremoris*, *S. aureus*, *B. subtilis*) by placing it onto a BPY medium plate and applying it evenly using a triangular spreader. Using the same method, 0.2 mL of yeast suspension was evenly applied onto the PDA medium. After the culture medium absorbed the bacterial solution, the culture dish was inverted, and 0.2 g or 0.4 g of whole or broken leaves from the test plant were added to the lid of the dish, respectively. A culture dish without added leaves served as the control and each group was tested in triplicate (the leaves did not contact the culture medium). The bacterial group was cultured in a 37°C incubator for 24 hours, while the yeast group was cultured in a 28°C incubator for 48 hours. The growth and colony size of bacteria on the culture plate medium were observed, and the number of colonies was calculated and compared with the control to calculate the antibacterial inhibition rate:


$$\text{Antibacterial inhibition rate}=\frac{\text{N}2-\text{N}1}{\text{N}2}\times 100\%$$

where N1 is the number of colonies in the treatment medium, and N2 is the number of colonies in the control medium.

### 2.4 Determination of BVOCs from bamboo leaves

Bamboo leaves from the sampling site with the strongest inhibition effect were selected to determine the BVOC composition using headspace solid-phase microextraction combined with gas chromatography-mass spectrometry (SPME-GC-MS). Bamboo leaves were collected and processed as described in 1.3.3. A mass of 1.00 g of sample was weighed and placed into a 20 mL headspace injection vial.

The extraction conditions were set at a constant temperature of 50°C, adsorption time of 40 min, equilibration time of 20 min, and resolution time of 5 min. The chromatographic column used was a DA-5MS (30 m × 0.25 mm (ID) × 0.25 µm).

Warming program: 40°C initial temperature, held for 3 min, heated to 120°C at a rate of 6°C·min^−1^, held for 3 min, heated to 250°C at a rate of 6°C·min^−1^, and then heated to 270°C at a rate of 10°C·min^−1^ and held for 5 min.

MS operating conditions: the temperature of the transmission line was 280°C; the temperature of the electron impact (EI) ion source was 280°C; the electron bombardment energy was 70 eV; the full scan range was 45~500 m/z. The results were analyzed using TurboMass Ver 5.4.2 version software and searched by the NIST 2.0 mass spectrometry database, and the volatile compositions were determined by referring to the relevant literature.

### 2.5 Data analysis

Data were analyzed using SPSS 19.0 (SPSS Inc., Chicago, IL, USA) for Windows. Two-way ANOVA and Student’s t-test were used to compare the means of different treatments for each data set at the significance level of *p*< 0.05 and at an extreme significance level of *p*< 0.01.

## 3 Results

### 3.1 Antibacterial results of the bamboo forest environments

**Figure 1:**
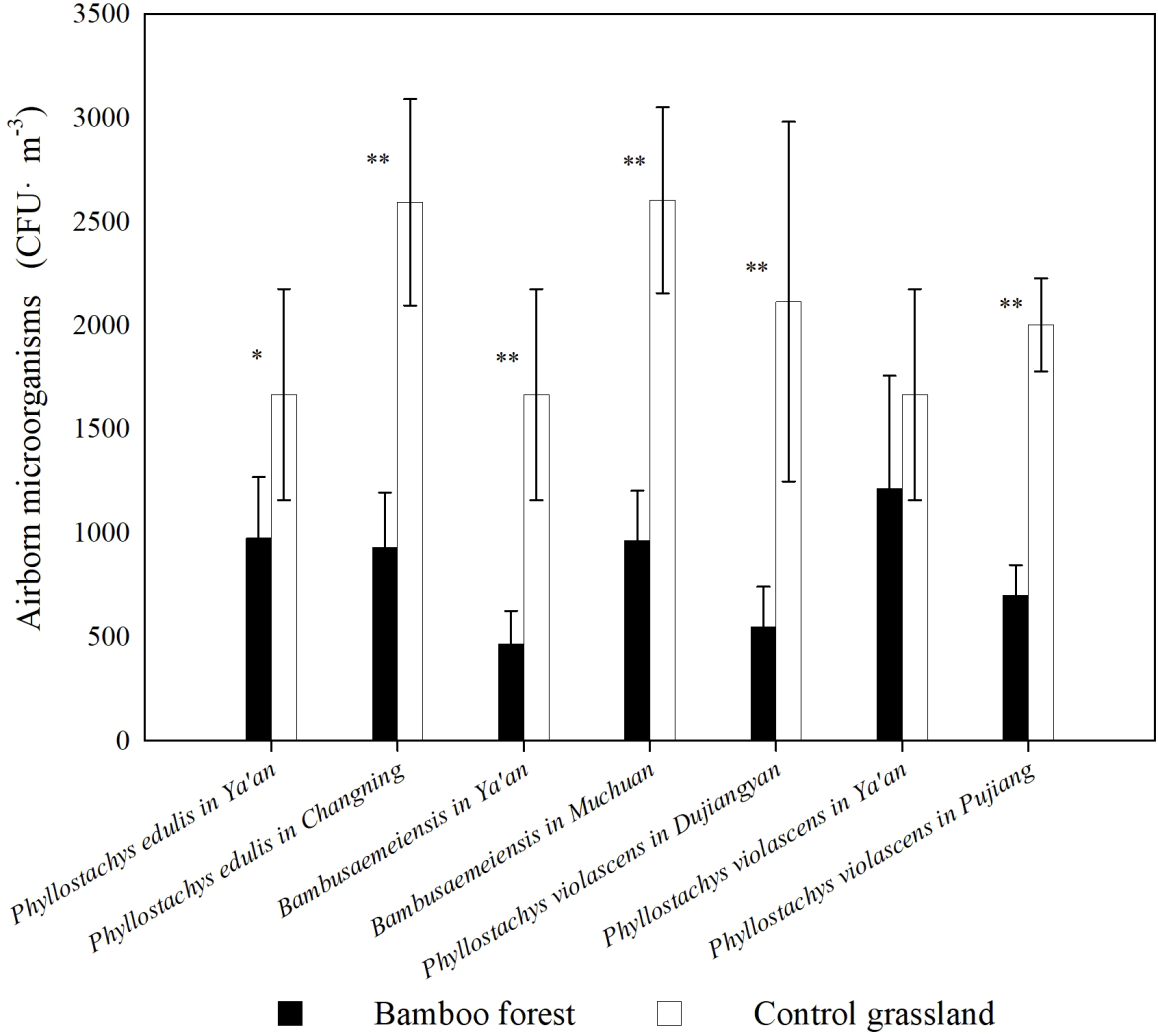
Microbial population of bamboo forest environments and adjacent grassland environments * represents a significant difference in microbial quantity between the bamboo forest environment and urban environment (*p* < 0.05), and ** represents an extremely significant difference in microbial quantity between the bamboo forest environment and urban environment (*p* < 0.01).

**Figure 2:**
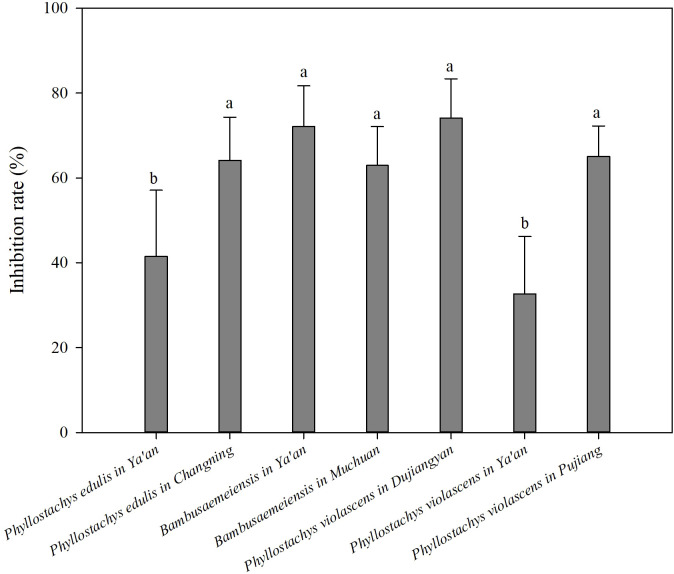
Comparison of bacterial inhibition rates at different bamboo forest sampling sites. Different lowercase letters represent three different bamboo species with significant differences in bacteriostasis rates (*p<* 0.05).

The results of this study showed that bamboo forests have a significant inhibitory effect on environmental microorganisms. Except for the *P. violascens* forest in Ya’an, the airborne microbial counts in the bamboo forests were significantly decreased compared with the urban control environment (*p*< 0.05, **Figure 1**). There were differences in the inhibition rates of each bamboo forest. The *P. violascens* forest in Dujiangyan had the highest inhibition rate of 74.14%, which was 2.27 times higher than the *P. violascens* forest in Ya’an, which had the lowest inhibition rate. **Figure 2**

### 3.2 Inhibitory effect of three species of bamboo leaves on gram-negative bacteria in *ex vivo* conditions

The results of the bacteriostatic test results on bamboo leaves against two Gram-negative bacteria showed that the three bamboo species had an inhibitory effect on both bacteria, and the difference in bamboo species had a small effect on this inhibitory effect. Only in the 0.4 g treatment group was the inhibition of *E. coli* by *B. emeiensis* leaves significantly higher than that of *P. edulis* and *P. violascens*, and the other treatments did not differ significantly. The bacterial inhibition effect of fragmented leaves from the same bamboo species was significantly higher than that of whole leaves, and a greater leaf mass induced a stronger bacterial inhibition effect (**Table 2**). There were significant differences in the tolerance of the two Gram-negative bacteria to bamboo leaf BVOCs, with *Shigella Castellani* being significantly less tolerant than *E coli* to *P. edulis* but more tolerant than *E. coli* to *B. emeiensis* and *P. violascens*.

**Table 2:** Inhibition of two Gram-negative bacteria by different treatments of bamboo leaves from three bamboo species.

Bamboo species	Bacteriostasis rate (%)							
	*Escherichia coli*				*Shigella Castellani*			
	0.2 g		0.4 g		0.2 g		0.4 g	
	whole leaves	fragmented leaves	whole leaves	fragmented leaves	whole leaves	fragmented leaves	whole leaves	fragmented leaves
*Phyllostachys edulis*	12.56aB	15.59aB	25.18bB	32.69bB	17.24aA	24.81aA	28.47aA	35.51aA
*Bambusa emeiensis*	11.29aA	22.78aA	30.96aA	41.93aA	11.73aA	16.87aB	24.38aB	33.7aB
*Phyllostachys violascens*	13.49aA	17.51aA	24.82bA	32.42bA	10.4aB	17.19aB	22.21aB	27.68aB

Different lowercase letters represent significant differences in the bacteriostatic effect of different bamboo species under the same treatment conditions (*p<* 0.05), and different uppercase letters represent significant differences in the inhibitory effect of the same bamboo species under different treatment conditions on the two Gram-negative bacteria (*p<* 0.05).

For the same bamboo species, the bacteriostatic effect of bamboo leaves varied depending on the sampling site. The inhibition rate of *P. edulis* 0.2 *g* collected from Changning against *E. coli* was 2.28 times higher than that collected from Ya’an; however, the inhibition rate against *Shigella Castellani* was only 44.28% of that from Ya’an. The inhibition rate of *P. violascens* for *Shigella Castellani* was significantly higher in bamboo leaves from Dujiangyan than from Ya’an and 19.08 times higher in the 0.2 g whole-leaf treatment. However, the difference in inhibition rate between *P. violascens* leaves from Dujiangyan and Ya’an against *E. coli* was not significant (**Tables 3** , **4**). This suggests that in addition to differences in bamboo species, the growth status of the plant may also affect the composition and release rate of BVOCs.

**Table 3:** Inhibition of *Escherichia coli* by three species of bamboo leaves.

Bamboo species	Location	Bacteriostasis rate (%)			
		0.2 g whole leaves	0.2 g fragmented leaves	0.4 g whole leaves	0.4 g fragmented leaves
*Phyllostachys edulis*	Ya’an	7.51cC	9.56eC	25.60aB	33.79bA
	Changning	17.36aD	21.49cbC	24.38aB	31.00bA
*Bambusa emeiensis*	Ya’an	11.33bcC	27.73aB	28.91aB	44.92aA
	Muchuan	10.26bcD	17.95cdC	33.33aB	38.46abA
*Phyllostachys violascens*	Dujiangyan	14.5abD	16.03deC	25.57aB	36.64abA
	Ya’an	11.42bcD	14.19deC	23.18aB	26.64cA
	Pujiang	14.1abD	23.20bC	25.71aB	37.3abA

Different lowercase letters represent significant differences in the bacteriostatic effect of different bamboo leaves under the same treatment conditions in different regions (*p<* 0.05), while different uppercase letters represent significant differences in the bacteriostatic effect between different treatments in the same region (*p<* 0.05). The table below displays equivalent information for different bacteria species.

**Table 4:** Inhibition of *Shigella Castellani* by three species of bamboo leaves.

Bamboo species	Location	Bacteriostasis rate (%)			
		0.2 g whole leaves	0.2 g fragmented leaves	0.4 g whole leaves	0.4 g fragmented leaves
*Phyllostachys edulis*	Ya’an	23.53aD	27.81aC	32.62bB	36.90bA
	Changning	10.42bC	22.01bB	23.55cdB	33.58bA
*Bambusa emeiensis*	Ya’an	11.11bD	16.46bC	20.58dB	36.21bA
	Muchuan	12.38bD	17.33bC	28.22bcB	31.19bA
*Phyllostachys violascens*	Dujiangyan	20.42aC	29.93aB	45.07aA	45.07aA
	Ya’an	1.07cD	2.86cC	3.57eB	6.07cA
	Pujiang	9.64bC	17.77bB	18.78dB	31.98bA

Different lowercase letters represent significant differences in the bacteriostatic effect of different bamboo leaves under the same treatment conditions in different regions (p < 0.05), while different uppercase letters represent significant differences in the bacteriostatic effect between different treatments in the same region (p < 0.05).

### 3.3 Inhibitory effect of bamboo leaves from three species on Gram-positive bacteria in *ex vivo* conditions

Differences in bacteriostatic effects on Gram-positive bacteria were found among the bamboo species. Interestingly, the differences in bacteriostatic effects of bamboo leaves subjected to fragmentation treatment varied, and it is possible that the fragmentation treatment of bamboo leaves from different bamboo species caused differing effects on their BVOC release. Specifically, among the *S. cremoris* inhibition treatments, *B. emeiensis* had the best inhibition effect using the whole-leaf treatment with an inhibition rate of 28.94%, which was significantly higher than *P. edulis* and *P. violascens*. However, the inhibition effect of *P. edulis* was significantly higher than *B. emeiensis* when using the leaf fragmentation treatment.

In the *S. aureus* inhibition treatment, the inhibition rate of *P. violascens* was significantly higher than the other two bamboo species, and no significant difference in the inhibition of *B. subtilis* between the three bamboo species was observed (**Table 5**).

**Table 5:** Inhibition of Gram-positive bacteria by different treatments of bamboo leaves from three bamboo species.

Bamboo species	Bacteriostasis rate (%)											
	*Staphylococcus cremoris*				*Staphylococcus aureus*				*Bacillus subtilis*			
	0.2 g whole leaves	0.2 g Fragmented leaves	0.4 g whole leaves	0.4 g Fragmented leaves	0.2 g whole leaves	0.2 g Fragmented leaves	0.4 g whole leaves	0.4 g Fragmented leaves	0.2 g whole leaves	0.2 g Fragmented leaves	0.4 g whole leaves	0.4 g Fragmented leaves
*Phyllostachys edulis*	5.80aB	11.04aC	19.79bB	41.35aA	7.89 bA	16.99abA	21.72abA	28.27aB	7.14aA	12.01aB	13.91aC	23.95aC
*Bambusa emeiensis*	7.03aB	16.40aB	28.94aA	35.42bA	7.82 bB	11.04bC	14.51bA	24.84aC	12.69aA	19.49aA	24.42aB	31.34aB
*Phyllostachys violascens*	8.55aC	15.19aB	19.56bB	23.53cC	16.70aA	23.18aA	25.81aA	34.10aB	10.87aB	22.10aA	18.68aB	37.31Aa

Different lowercase letters represent significant differences in the bacteriostatic effect of different bamboo species under the same treatment conditions (*p<* 0.05), while different uppercase letters represent significant differences in the inhibitory effect of the same bamboo species under different treatment conditions on the three Gram-positive bacteria (*p<* 0.05).

Similar to the inhibition results of Gram-negative bacteria, the inhibitory effect of bamboo leaves on Gram-positive bacteria increased significantly with an increase in the degree of fragmentation and mass, with the highest inhibition rate in the 0.4 g fragmented leaves treatment group. The inhibition rate of the *P. edulis* 0.4 g fragmented leaves treatment against *S. cremoris* was up to 12.33 times that of the 0.2 g whole-leaf treatment.

Differences in sampling sites also affected the inhibitory effect of Gram-positive bacteria (**Tables 6**–**8**). Significant differences in the inhibition of *P. edulis* among different sampling sites were only observed in the 0.4 g of leaves treatment against *S. cremoris*. *B. emeiensis* from different locations showed significant differences against *B. subtilis* and *Shigella Castellani*, with 0.2 g of fragmented leaves and 0.4 g of whole leaves. Leaves from the three *P. violascens* sampling sites showed significant differences in all treatments except for 0.2 g of fragmented leaves against *S. cremoris*.

**Table 6:** Inhibition of *Staphylococcus cremoris* by three species of bamboo leaves.

Bamboo species	Location	Bacteriostasis rate (%)			
		0.2 g whole leaves	0.2 g fragmented leaves	0.4 g whole leaves	0.4 g fragmented leaves
*Phyllostachys edulis*	Ya’an	7.95bcC	11.93aB	12.50cB	38.07bA
	Changning	3.63cdD	10.08aC	27.42aB	44.76aA
*Bambusa emeiensis*	Ya’an	7.27bcD	18.18aC	28.18aB	34.09bA
	Muchuan	6.72bcD	14.18aC	29.48aB	36.57bA
*Phyllostachys violascens*	Dujiangyan	9.79bC	17.02aB	19.15bB	26.38cA
	Ya’an	13.79aD	18.28aC	25.52aB	27.59cA
	Pujiang	2.02dD	9.76aC	14.14bcB	16.84dA

Different lowercase letters represent significant differences in the bacteriostatic effect of different bamboo leaves under the same treatment conditions in different regions (p < 0.05), while different uppercase letters represent significant differences in the bacteriostatic effect between different treatments in the same region (p < 0.05).

**Table 7:** Inhibition of *Staphylococcus aureus* by three species of bamboo leaves.

Bamboo species	Location	Bacteriostasis rate (%)			
		0.2 g whole leaves	0.2 g fragmented leaves	0.4 g whole leaves	0.4 g fragmented leaves
*Phyllostachys edulis*	Ya’an	6.72cdD	17.23bcC	22.27bB	31.09bA
	Changning	9.21bcD	16.83bcC	21.59bB	25.71bA
*Bambusa emeiensis*	Ya’an	7.49cD	13.86cC	17.23bcB	25.09bA
	Muchuan	3.68cdD	7.89dC	11.58dB	24.21bA
*Phyllostachys violascens*	Dujiangyan	13.03bC	22.18bB	22.89bB	26.06bA
	Ya’an	1.51dD	11.56cdC	15.08bcB	29.15bA
	Pujiang	26.91aD	34.94aC	39.76aB	47.79aA

Different lowercase letters represent significant differences in the bacteriostatic effect of different bamboo leaves under the same treatment conditions in different regions (p < 0.05), while different uppercase letters represent significant differences in the bacteriostatic effect between different treatments in the same region (p < 0.05).

**Table 8:** Inhibition of *Bacillus subtilis* by three species of bamboo leaves.

Bamboo species	Location	Bacteriostasis rate (%)			
		0.2 g whole leaves	0.2 g fragmented leaves	0.4 g whole leaves	0.4 g fragmented leaves
*Phyllostachys edulis*	Ya’an	7.22bcD	11.79bC	14.83bB	22.05cdA
	Changning	6.81bcC	11.91bB	12.77bB	25.53cA
*Bambusa emeiensis*	Ya’an	9.39bcD	12.27bC	16.61bB	19.13cdA
	Muchuan	16.49aD	26.80aC	31.96aB	43.30bA
*Phyllostachys violascens*	Dujiangyan	12.57abD	28.96aB	14.75bC	54.64aA
	Ya’an	2.54cC	10.87bB	11.59bB	13.77dA
	Pujiang	18.60aD	27.91aC	30.23aB	44.19bA

Different lowercase letters represent significant differences in the bacteriostatic effect of different bamboo leaves under the same treatment conditions in different regions (p < 0.05), while different uppercase letters represent significant differences in the bacteriostatic effect between different treatments in the same region (p < 0.05).

### 3.4 Inhibitory effect of bamboo leaves from three species on yeast in *ex vivo* conditions

Under the same treatment conditions, *P. edulis* inhibited yeast significantly more than the other two bamboo species (**Table 9**). The inhibition rate of *P. edulis* was 2.05 and 3.52 times higher than that of *B. emeiensis* and *P. violascens* under the treatment of 0.4 g of fragmented leaves, respectively. The difference between *B. emeiensis* and *P. violascens* in terms of the inhibition of yeasts was not significant.

**Table 9:** Inhibition of *Candida albicans* by bamboo leaves from three bamboo species.

Bamboo species	Bacteriostasis rate (%)			
	*Candida albicans*			
	0.2 g whole leaves	0.2 g fragmented leaves	0.4 g whole leaves	0.4 g fragmented leaves
*Phyllostachys edulis*	22.28aC	33.58aB	35.61aB	45.05aA
*Bambusa emeiensis*	6.89bD	11.73bC	16.81bB	21.99bA
*Phyllostachys violascens*	9.46bA	10.4bA	12.28bA	12.81bA

Different lowercase letters represent significant differences in the bacteriostatic effect of different bamboo leaves under the same treatment conditions in different regions (p < 0.05), while different uppercase letters represent significant differences in the bacteriostatic effect between different treatments in the same region (p < 0.05).

Differences in sampling sites for the same bamboo species had a significant effect on the inhibitory effect on yeast (**Table 10**). The inhibition rate of *P. edulis* leaves collected in Ya’an was significantly higher than in Changning, and the inhibition rate of *B. emeiensis* leaves collected in Ya’an was also significantly higher than in Muchuan.

**Table 10:** Inhibitory effect of bamboo leaves from different sampling sites on *Candida albicans*.

Bamboo species	Location	Bacteriostasis rate (%)			
		0.2 g whole leaves	0.2 g fragmented leaves	0.4 g whole leaves	0.4 g fragmented leaves
*Phyllostachys edulis*	Ya’an	26.22aC	43.71aB	45.10aB	52.45aA
	Changning	18.06bC	23.61bB	26.39bB	37.50bA
*Bambusa emeiensis*	Ya’an	12.04bcD	18.52bcC	26.39bB	29.63cA
	Muchuan	1.09dD	4.92dC	7.10dB	14.75dA
*Phyllostachys violascens*	Dujiangyan	9.56cA	10.29cA	12.50cA	12.87dA

Different lowercase letters represent significant differences in the bacteriostatic effect of different bamboo leaves under the same treatment conditions in different regions (p < 0.05), while different uppercase letters represent significant differences in the bacteriostatic effect between different treatments in the same region (p < 0.05).

### 3.5 BVOCs composition in *P. edulis* leaves from Changning

**Figure 3:**
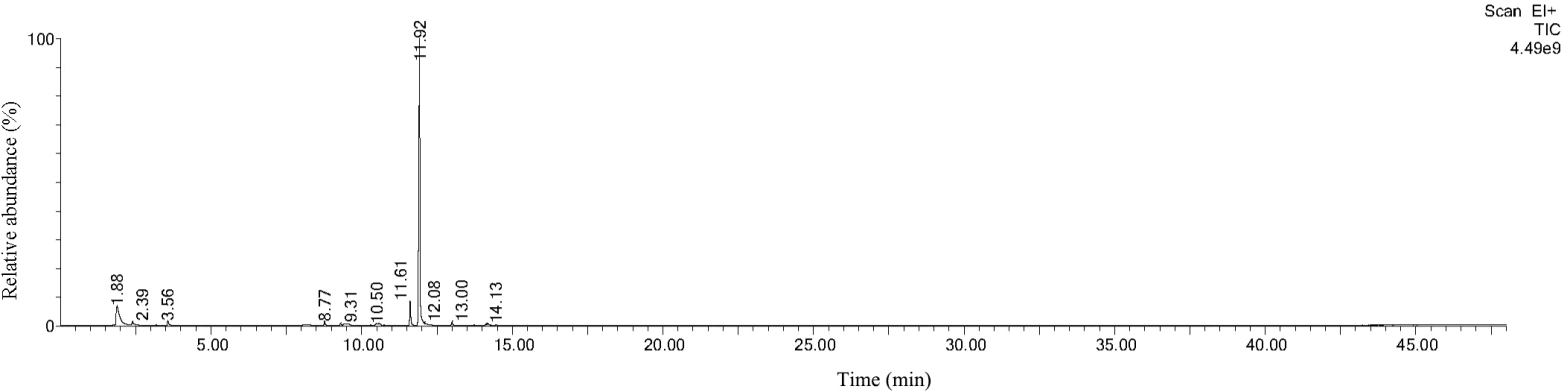
Total ion chromatoGram of BVOCs in the leaves of *Phyllostachys edulis* collected in Changning by using headspace SPME-GC/MS.

*P. edulis* collected in Changning showed a relatively high antibacterial rate against six tested microorganisms and the highest proportion of terpenes was found among the BVOCs in its leaves. Terpenes comprised 63% of all measured BVOCs; ocimene was the most abundant BVOC component in *P. edulis* from Changning. **Figure 3**

## 4 Discussion

### 4.1 Antibacterial effects of bamboo forests are influenced by the growing conditions

The type and rate of BVOCs plants released is related to the plant’s ability to inhibit bacteria (Farmer, 2001; Sartorelli et al., 2007). Some BVOCs have strong antimicrobial effects, such as terpenes and aldehydes (Dorman and Deans, 2000). Environmental factors can also directly affect the release of plant BVOCs (Tyagi and Malik, 2011; Eller et al., 2016; Effah et al., 2020), and among the various environmental factors light and temperature are the key factors affecting BVOCs release (Staudt and Lhoutellier, 2011; Lindwall et al., 2015; Yu et al., 2021). In order to make the temperature and light intensity as similar as possible, we all chose to take samples in sunny and windless weather from July to August in Sichuan Province. The results showed that, except for the artificially planted nursery (P. violascens forest in Ya’an), the number of airborne microorganisms in bamboo forests was significantly lower compared with those in grasslands in the same area (control treatment), and significant differences were observed in antimicrobial capacity among different sampling sites for the same bamboo species. The varieties and composition of BVOCs are chemically diverse by the plant species and circumstances in which the plants grow (Iijima, 2014). Therefore, the antimicrobial capacity of the same bamboo species varies in different sampling sites, which is similar to the antimicrobial results of essential oil of bamboo leaves (Jin et al., 2011). The P. violascens in Ya’an were planted in nurseries, and the age of the bamboo was only eight years, so the growing environment and growth were not as good as other naturally growing bamboo forests, and the antimicrobial capacity was significantly lower than others. Furthermore, the differences in the inhibition rates of the natural bamboo forests were not significant.

### 4.2 BVOCs spontaneously released from bamboo leaves are more effective in inhibiting Gram-negative bacteria than Gram-positive bacteria

In the present study, the inhibitory ability of spontaneously released BVOCs from bamboo leaves against Gram-negative bacteria was generally stronger than that of Gram-positive bacteria, and was particularly significant in the 0.2 g treatment group. This is similar to the results of the bacterial inhibition of the essential oil of bamboo leaf (Tao et al., 2019). BVOCs are hydrophobic, which allows them to penetrate the lipids of bacterial cell membranes, disrupting the structure, and making it more permeable (Sikkema et al., 1994). This inhibits bacterial growth and even leads to death (Denyer and Hugo, 1991). The cell walls of Gram-negative bacteria are thinner than those of Gram-positive bacteria, and are more sensitive to BVOCs.

### 4.3 Antimicrobial activity of bamboo BVOCs showed strain specificity 

*P. edulis*, *B. emeiensis*, and *P. violascens* in this study belong to the Bambusoideae (Poaceae) subfamily, but the antibacterial capacity differed significantly. It has been suggested that species differences are the main reason for the difference in BVOCs release (Llusia et al., 2002; Iijima, 2014), thus affecting antimicrobial capacity. Interestingly, the strength of inhibition of bamboo leaf BVOCs was strain-dependent. For example, the BVOCs released from *B. emeiensis* leaves were weakly inhibitory to bacteria compared to other bamboo species, but strongly inhibitory to yeasts, significantly higher than from bamboo leaves collected in the other bamboo forests. Also, *P. edulis* leaves collected in Ya’an inhibited *S. cremoris* significantly less than those collected in Changning, however, they inhibited *C. albicans* significantly more than those of *P. edulis* from Changning. Similar results were observed when bamboo leaves were cut up. This result is similar to the antimicrobial results of essential oils of different bamboo species (Jin et al., 2011). Studies on the antibacterial activity of BVOCs mainly focused on BVOCs essential oils, and it was concluded that essential oils with aldehydes or phenols as their main components had the strongest antimicrobial activity, followed by terpene alcohols (Anselmo-Moreira et al., 2021; Jin et al., 2011; Dhifi et al., 2016). However, the specific composition of BVOCs with strong inhibitory ability to specific microorganisms needs to be further investigated.

Growth condition is an important factor influencing the release of BVOCs, and differences in environmental conditions such as light, temperature, humidity and soil conditions in the sampling sites resulted in different plant growth and composition of BVOCs (Jin et al., 2011). *P. edulis* in Changning is a natural bamboo forest with a large area and a good natural ecological environment, which results in vigorous growth of *P. edulis* and strong antibacterial activity in *ex vivo* conditions. In addition, although all the leaves collected in this experiment were mature leaves, the bamboo forests had undergone different growing times, so the seedling ages of the collected bamboo also differed, resulted in differing releases of BVOCs from the same bamboo species (Bai et al., 2016; Motonori et al., 2018; Tang et al., 2023). The overall difference in the inhibitory ability of *P. edulis* and *B. emeiensis* collected in Ya’an was not significant, and it was hypothesized that the growth status of the bamboo was the main influencing factor on the inhibitory ability.

### 4.4 With Ocimene is the main BVOCs component, *P. edulis* is an excellent bamboo species for forest recreation

Bamboo’s volatile organic compounds possess health benefits (Guo et al., 2015). In this study, the main component of *P. edulis* BVOCs with the strongest overall inhibitory capacity was terpenes.Terpenoids are common constituents of BVOCs in plants and are especially released as signal substances for defense mechanisms when plants are injured (Kroes et al., 2017; Vivaldo et al., 2017). Studies have shown that most terpenoids have strong antibacterial properties (Senatore, 2000). Besides, terpenes have shown health benefits. For example, monoterpenes increases α-waves and decreases β-waves in brain, inducing a state of relative sedation (Linck et al., 2010; Kasuya et al., 2015). The BVOCs composition of *P. edulis* is safe and can be used not only for forest healing but also has the potential to be applied as a biologically active antimicrobial agent.

It has been shown that alcohols and acids are the main components of the essential oil of *P. edulis* (Jin et al., 2011; Tao et al., 2019), while terpenoids is the main component of BVOCs in this study. It indicated that BVOCs spontaneously released from bamboo leaves are highly reactive chemical species, which differ from the composition of essential oils. For forest recreation, volatile gases are the main active substances with healing effect. More attention should be paid to the analysis of volatile constituents in the study of forest recreation.

## 5 Conclusion

BVOCs from bamboo plants exhibit strong antibacterial properties that vary depending on the environmental conditions of the bamboo forest and the growth status of the plants. Generally speaking,the inhibitory ability of BVOCs from bamboo leaves against Gram-negative bacteria was generally stronger than that of Gram-positive bacteria. Antimicrobial activity of bamboo BVOCs showed strain specificity and *B. emeiensis* leaves collected in Ya’an show strong antibacterial ability against yeast, and *P. violascens* show strong antibacterial ability against *Staphylococcus aureus* and *Bacillus subtilis*. The *ex vivo* leaves of *P. edulis* collected in Changning possess stronger antibacterial activity against yeast, *S. cremoris*, and *Shigella Castellani* than the other sampled bamboo leaves. Additionally, the BVOCs of *P. edulis* leaves primarily comprise beneficial terpenes, making it a good choice for recreational bamboo forests.CRediT authorship contribution statement

## Data Availability

The original contributions presented in the study are included in the article/supplementary material. Further inquiries can be directed to the corresponding author.
